# Treatment of myofascial pain syndrome with lidocaine injection and physical therapy, alone or in combination: a single blind, randomized, controlled clinical trial

**DOI:** 10.1186/s12891-016-0949-3

**Published:** 2016-02-24

**Authors:** Luz Helena Lugo, Hector Ivan García, Heather L. Rogers, Jesús Alberto Plata

**Affiliations:** Health Rehabilitation Group and Academic Group of Clinical Epidemiology, University of Antioquia, Carrera 53 # 61-30, Medellín, Antioquia Colombia, South America; Department of Methodology and Experimental Psychology, University of Deusto, Avda. De las Universidades, 24, Bilbao, Spain

**Keywords:** Myofascial pain, Trigger points, Lidocaine injection, Physical therapy

## Abstract

**Background:**

Myofascial pain syndrome (MPS) of the shoulder girdle and cervical region is a common musculoskeletal problem that is often chronic or recurrent. Physical therapy (PT) and lidocaine injections (LI) are two treatments with demonstrated effectiveness compared to a control group, however little is known about their combined value. The objective of this study was to determine whether LI into trigger points combined with a PT program would be more effective than each separate treatment alone in improving pain, function, and quality of life in a group of patients with MPS of the shoulder girdle and cervical region.

**Methods:**

A single-blind, randomized, controlled clinical trial (RCT) was conducted with three parallel groups in the Departments of Physical Medicine and Rehabilitation of two urban hospitals in Medellin, Colombia. One hundred and twenty seven patients with shoulder girdle MPS for more than 6 weeks and pain greater than 40 mm on the visual analog scale (VAS) were assigned to 1 of 3 intervention groups: PT, LI, or the combination of both (PT + LI). The primary outcome was VAS pain rating at 1-month post-treatment. The secondary outcomes included VAS pain rating at 3 months, and, at both 1 and 3 months post-treatment: (a) function, evaluated by hand-back maneuver and the hand-mouth maneuver, (b) quality of life, as measured by sub-scales of the Short Form – 36 (SF-36), and (c) depressive symptoms, as measured by the Patient Health Questionnaire – 9 (PHQ-9). Independent t-tests were used to compare outcomes between groups at 1 month and 3 months post-treatment.

**Results:**

In the per protocol analysis, there were no significant intergroup differences in VAS at 1 month PT + LI, 40.8 [25.3] vs. PT, 37.8 [21.9], *p* = 0.560 and vs. LI, 44.2 [24.9], *p* = 0.545. There were also no differences between groups on secondary outcomes except that the PT and PT + LI groups had higher right upper limb hand-back maneuver scores compared to the LI alone group at both 1 and 3 months (*p* = 0.013 and *p* = 0.016 respectively).

**Conclusions:**

The results of this RCT showed that no differences in pain ratings were observed between the individual treatments (PT or LI) compared to the combined treatment of PT and LI. In general, no difference in primary or secondary outcomes was observed between treatments.

**Trial registration:**

NTC01250184 November 27, 2010.

## Background

Myofascial pain syndrome (MPS) is a major cause of chronic musculoskeletal pain and is characterized by the presence of hypersensitive areas and myofascial trigger points (MTrPs) in a muscle or its fascia that, when palpated, may trigger a characteristic referred pain, tenderness and autonomic phenomena [1].

The prevalence of MPS varies from 21 % in orthopedic clinics to 30 % in general practice, and between 55 and 95 % in specialized pain management centers [2, 3]. Some studies suggest that latent MTrPs may be present in between 45 and 55 % of the muscles of the shoulder girdle and between 5 and 55 % of the muscles of the pelvic girdle of asymptomatic young adults [4]. Myofascial pain syndrome can be acute or chronic and primary or secondary. MPS may be associated with radiculopathy, nerve entrapments, metabolic and nutritional disorders, biomechanical imbalance, and/or physical deconditioning [5].

The concept of MTrPs can be considered to be controversial because some researchers argue that the key phenomenon of muscle tenderness demands a robust plausible explanation based on neurobiology [6]. In response, other researchers purport this argument is not sufficient to invalidate the integrated trigger point hypothesis [7]. They insist that numerous physiological mechanisms have been proposed. The integrated trigger point hypothesis is the most accepted physiological model to explain these phenomena. This model explains how abnormal depolarization of the post-junctional membrane of motor endplates causes a localized hypoxic energy crisis associated with sensory and autonomic reflex arcs that are sustained by complex sensitization mechanisms [8, 9]. Biochemical abnormalities have been described to explain this neuromuscular dysfunction consisting of motor and sensory abnormalities involving both the peripheral and central nervous system [10, 11]. A more recent hypothesis suggests that MTrPs are caused by a nociception-induced central nervous system disorder which is centrally maintained by α-motoneuron plateau depolarization, but unfortunately experimental evidence for this hypothesis is still lacking [12, 13]. Furthermore, it is possible that some of the mechanisms proposed to explain chronic non-specific low back pain could be considered to apply to chronic myofascial pain in the cervical region [14]. For example, psychosocial factors may make acute pain become chronic and, indeed, patients with MPS have been found to have higher rates of anxiety and depression [15].

The diagnosis of MPS can also be considered controversial. Some researchers suggest that focal areas of muscle pain defined as associated with MTrPs cannot be reliably identified. Although inter-observer variability does exist, research shows that, after implementing a training program to ensure systematic physical examinations, an appropriate percentage of agreement between different examiners can be achieved, especially when palpating the trapezius, gluteus maximus and quadratus lumborum trigger points [16–18]. Various methods have been utilized to achieve better reproducibility of the diagnosis of MPS. For instance, Simons and Travell’s method takes into account the existence of MTrPs during muscle palpation, a characteristic pattern of referred pain, the display of local response contractions or twitches, and joint mobility restriction in compromised muscles. Functional limitation is evaluated by means of specific movements such as the hand-back (patient’s ability to touch the lower angle of the contralateral scapula) and the hand-mouth (patient’s ability to exceed the contralateral labial commissure when passing the hand behind the head) maneuvers [1, 19].

There are many different treatments for MPS. Non-invasive treatments include therapeutic exercise, ultrasound, and massage and stretching the affected muscle with aerosolized fluoromethane. A systematic review of 21 randomized controlled trials (RCTs) [20] showed moderate quality evidence according to the GRADE (Grading of Recommendations Assessment, Development, and Evaluation) system [21] that stretching and strengthening are effective for improving pain, function, and perception of improvement in chronic neck pain in the early and intermediate follow-up periods. In another systematic review of 26 RCTs, exercise proved to be effective for improving pain and function in rotator cuff disease [22]. A meta-analysis synthesizing the evidence from 12 studies indicated that high-quality therapeutic massage was effective for neck and shoulder pain, but did not improve function [23]. No interventions have demonstrated the effectiveness of capsaicin patches [24], except those with lidocaine, which improved pain over a 1-month period [25].

Invasive treatments have also been used to treat MPS, including dry-needling or medications, such as local anesthetics, corticosteroids, or botulinum toxin [5]. Dry needling has proven effective, with improvement depending on the technique used [26]. Lidocaine and bupivacaine injections were found to be more effective than placebo injections for improving pain [25, 26], with the needle size used (21, 23, and 25 gauge) unrelated to treatment effectiveness [27]. Studies of non-steroidal anti-inflammatory drugs (NSAIDs), psychotropic medications, steroid injections, and muscle relaxants have been conducted with limited evidence and unclear benefits [28, 29].

## Methods

### Aim

Because of the lack of research examining which treatments, or combination of treatments, are more effective for MPS, the objective of the present study was to determine whether lidocaine injection (LI) into MTrPs combined with a physical therapy (PT) program was more effective than each separate treatment alone on the outcomes of pain, function, and quality of life in a group of patients with MPS of the shoulder girdle and cervical region.

### Study design and setting

A single blind, randomized, controlled clinical trial (RCT) was conducted in two different city hospitals in Medellín, Colombia (IPS Universitaria and Clínica Las Americas) between January 2009 and October 2011. Approval was obtained from the Ethics Committee of the University of Antioquia.

### Participants

Using the patient databases of two general and specialized medical institutions, 762 patients were identified to have non-specific myalgia or muscle disorders diagnoses. These diagnoses were chosen because the International Classification of Disease Version 10 (ICD-10) does not have a specific code for MPS. The inclusion/exclusion criteria were: 1. To be older than 18 years of age. 2. To have MTrPs in one or more of the following muscles: the trapezius, the infraspinatus, and/or the levator scapulae (cervical portion), diagnosed by neck or shoulder pain, which may or may not be accompanied by the typical pattern of referred pain in the compromised muscle. 3. To have had neck or shoulder pain over the prior 6 weeks (because there is controversy concerning the cutoff for acute pain as either 4 or 6 weeks, 6 weeks was chosen). 4. To have a minimum pain score of 40 mm on the visual analog scale (VAS). 5. To not have moderately severe depression, as indicated by a score of higher than 15 on the Patient Health Questionnaire (PHQ-9) measure of depressive symptoms, and/or presence of a psychiatric or bipolar affective disorder. 6. To not have comorbid conditions such as Fibromyalgia according to the criteria of American College of Rheumatology [30, 31], polyneuropathy, entrapment syndromes and radiculopathies of the upper limb, presence of inflammatory or infectious diseases that were not being medically treated, history of whiplash, or allergy to local anesthetics. 7. To not be receiving treatment or have received treatment in the last month. 8. To not be consuming painkillers or continuously using anti-inflammatory medications.

A nurse with 10 years of research experience conducted a phone screen of 762 patients to assess the inclusion and exclusion criteria. If the patients were older than 18 years of age, had neck pain or shoulder girdle pain, and had not received treatment in the last month, the patient was given an appointment for a medical evaluation. Two hundred and sixty seven patients were excluded during the phone screen for having a non-MPS diagnosis, MPS of less than 6 weeks duration, or in current treatment for MPS. Four hundred and ninety-five patients were given an appointment at the hospital for a clinical assessment. At the appointment, two medical physiatrists (LHL and JAP) with over 10 years of research experience performed a clinical evaluation of the MPS patients and reviewed their medical history in order to verify inclusion and exclusion criteria. At this stage, 360 patients were excluded due to: moderately severe depression with a score higher than 15 on the Patient Health Questionnaire (PHQ-9) measure of depressive symptoms and/or presence of psychotic or bipolar affective disorder and/or existence of comorbid conditions listed in the inclusion/exclusion criteria. The selected muscles (according to their anatomical location) were carefully palpated using a gel and manual detection of trigger points. Patients were observed and asked if they experienced pain. If they responded affirmatively, they were asked if that pain was located in the same palpation point or in a different point. In addition, patients were also asked to indicate the location of the pain on a form with drawings of the muscles and their trigger points.

All eligible patients were invited to participate in the study. The patients were informed of the study before the first assessment and signed a written, informed consent statement.

**Fig. 1:**
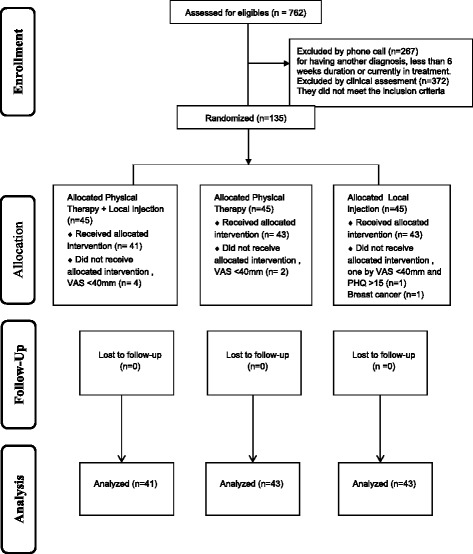
Flow diagram of participants

The final sample consisted of 135 patients, with 45 patients were randomly allocated to each of the three groups: Physical Therapy (PT) + Local Injection (LI), PT only, and LI only. After randomization, 2 patients in the PT + LI group were excluded (one of them with VAS < 40 mm and the other with PHQ-9 > 15), 4 patients in the PT group (for having VAS < 40 mm), and 2 patients in the LI group (one because of breast cancer and the other due to VAS < 40 and PHQ-9 > 15) (Fig. 1).

### Interventions

Patients were randomly assigned to 1 of the following 3 groups: group 1, the PT + LI group, included 41 patients who underwent a PT program and trigger point LI with 0.5 % lidocaine without epinephrine; group 2, the PT group, included 43 patients who underwent the PT program alone; and group 3, the LI group, included 43 patients who underwent trigger point LIs with 0.5 % lidocaine without epinephrine alone.

The PT intervention included 12 sessions of PT lasting 1 h, 3 times per week, for 4 weeks. The average number of PT sessions attended in the PT + IL group was 8.7 (SD: 4.7), while the average number of PT session attended in the PT group was 7.4 (SD: 5.2). The physical therapy intervention in both groups (PT and PT + LI) at each session consisted of 10 min of hot pack and ultrasound with 0.8 W/cm^2^ and 1 Hz. Then the physical therapist deactivated active MTrPs via manual compression for 10 min. The deactivation procedure required the physical therapist to apply gentle, gradually increasing pressure on the MTrPs until the finger encountered a definite increase in tissue resistance. The pressure was maintained until the therapist sensed relief of tension under the palpating finger or the patient experienced a considerable decline in pain. This technique was combined with other manual techniques, such as deep stroking (pressure directed along the length of the taut band) or strumming (pressure applied perpendicularly across the muscle fibers). Then muscle-stretching and strengthening exercises were performed for the cervical and shoulder girdle and specific for trapezius, infraspinatus, and the levator scapulae muscles for a total of 30 min, followed by exercises to improve joint range of motion for 10 min more. This was protocol was standardized by LHL based on various publications and recommendations [20, 22, 23] and conducted by one of three physiotherapists with more than 5 years of experience.

The LI intervention consisted of an injection of 0.5 % lidocaine without epinephrine into MTrPs. The injections were given as follows: once the MTrP was located, antisepsis was performed using 70 % alcohol, the MTrP was located between two fingers, and a 1-2 cm sterile needle with a thickness of 25 or 26 gauge was inserted into the MTrP at an angle of 30° with respect to the skin. To ensure that the needle was not in a blood vessel, the needle was aspirated before injecting a small amount (0.2 ml) of 0.5 % lidocaine. Then, the needle was withdrawn to the subcutaneous tissue and redirected superiorly, inferiorly, laterally and medially, repeating the process described above [5, 27]. Pressure was applied to the injected area to promote hemostasis, and adhesive tape was used to cover the skin. Patients were advised to gradually return to their activities, avoiding strenuous activities for the first 3 to 4 days after the injection. On the day that patients were randomly assigned to the PT + LI group, they received the LI intervention and 2 to 3 days later, they began the PT treatment.

If there was no improvement in pain, all patients were allowed to use analgesics, such as acetaminophen 2-3g/day and/or ibuprofen 1.2 to 2.4 g/day.

### Outcomes

The primary outcome was pain at 4 weeks after the day of initial evaluation. Pain was measured using a VAS with a score of 0–100, where 100 is the value representing the highest degree of pain. Successful treatment was defined as a reduction in pain of at least 20 % of the previous score on the VAS after 4 weeks of the initial evaluation, or a reduction of 14 mm on the VAS. The VAS is a reliable pain assessment measure that has been validated previously [29]. At each time of assessment, patients were asked about their pain in the last 24 h.

The following secondary outcomes were assessed at 1 and 3 months post-treatment: quality of life (QoL), depressive symptoms, and function. QoL dimensions, including bodily pain were assessed via the Short Form-36 (SF-36). The SF-36 consists of 36 items addressing the patient’s perception of their QoL in the following eight domains: physical function (PF), role limitations due to physical problems (RP), bodily pain (BP), general health (GH), vitality (VT), social functioning (SF), role limitations due to emotional problems (RE), and mental health (MH), and one item describing change in health. Sub-scale scores range from 0 to 100, with 100 as the best, most positive QoL in that area and 0 is the worst, this scale has been validated in Colombia [32]. Depressive symptoms were measured using the Colombian version of the Patient Health Questionnaire – 9 (PHQ-9). The PHQ-9 was developed from the Primary Care Evaluation of Mental Disorders (PRIME-MD) and evaluates each of the nine criteria for major depression according to the Diagnostic and Statistical Manual of Mental Disorders – IV (DSM-IV). The self-report instrument asks individuals how much they had been bothered by any of the nine problems over the prior two weeks. Items are scored from 0 (not at all) to 3 (nearly every day). Items are summed and the total score (from 0 to 27) represents the severity of depressive symptoms [33]. The Colombian version of the scale was used and is available by Pfizer. Function was evaluated using the hand-back maneuver (patient’s ability to touch the lower angle of the contralateral scapula) and the hand-mouth maneuver (patient’s ability to exceed the contralateral labial commissure when passing the hand behind the head) [1, 19]. Higher scores indicate better function. A final secondary outcome was pain rating at 3 months post-treatment measured using a VAS with a score of 0–100, where 100 is the value representing the highest degree of pain.

### Study procedures

#### Randomization

The randomization process was performed using permuted blocks (with RALLOCK software) to avoid an imbalance in the number of patients in each group and to leave open the possibility of an interim analysis, if necessary. This process was performed by an investigator (HIG) who had no contact with the patients. The allocations to each treatment were placed in consecutive sealed, opaque envelopes. They were kept in the research headquarters and were taken to the site according to the number of patients referred.

At the initial appointment, two Physical Medicine & Rehabilitation (PM&R) physician researchers (LHL, JAP) with over 5 years of experience in the treatment of MPS performed the clinical evaluation and noted the relevant information on the patient forms. A nurse then administered the VAS, SF-36, and PHQ-9 questionnaires, and evaluated the participant’s function. In addition, patients were asked if, over the prior month, they had experienced stressful situations and/or sleep disturbances. They were also asked for their occupation to assess the need to engage in continuous positions. The participant subsequently opened an opaque, sealed and sequentially numbered envelope to discover the group to which they were assigned. If the patient was in one of the two groups receiving injections, the physician researcher (LHL, JAP) immediately administered the injection per the intervention procedures described above. Those participants assigned to 1of the 2 PT groups received the dates and times of 12 appointments with a selected and trained physiotherapist. Outcomes were assessed at baseline and at 4 and 12 weeks and were evaluated by a trained nurse (RR) with more than 10 years of experience in the application of the instruments. The nurse was independent of the researchers and blind to the intervention.

#### Single blinding

The PM&R physicians who evaluated the patients at baseline and the nurse who assessed the patients at baseline and follow-up were blinded to treatment allocation. Physicians who performed the MTrPs injection did not participate in the evaluation of primary and secondary outcomes. Physical therapists who conducted the therapeutic exercise program did not participate in the evaluation of primary and secondary outcomes.

### Statistical analysis

#### Sample size

Sample size was calculated with the software “Sample Size from Javeriana University” [34], with the following parameters according to the literature review: type I error = 0.05, type II error = 0.2 (80 % power), number of measurements before randomization = 1, number of measurements performed after randomization = 2, correlation between measurements = 0.6, and clinically significant difference 0.35, or a reduction in pain of at least 20 % for an n of 45 patients per group [35].

#### Data analyses

The baseline data of each group prior to the intervention was described using frequency distributions and averages with standard deviations. The normality of the distributions of the quantitative variables was tested using the Kolmogorov-Smirnov test. Comparisons of baseline data were made between all three groups (PT+ IL vs. PT vs. IL at baseline using Kruskal-Wallis tests or chi square tests). Independent t-tests were used to comparison of outcomes between groups at 1 month and 3 months post-treatment. The baseline characteristics of the groups before starting the intervention and after randomization are shown in Table 1. Missing patients were not included in the data analysis. A per-protocol analysis was conducted.

**Table 1:** Base-line characteristics of the patients

Characteristics	Physical therapy + local injection	Physical therapy	Local injection	
	(*n* = 41)	(*n* = 43)	(*n* = 43)	*p*
Sex (%)				
Female	35 (85.4)	36 (83.7)	33 (76.7)	0.55*
Age (Mean, SE)	37.2 (11.1)	42.6 (9.7)	37.7 (11.8)	0.081**
Residence (%)				
Medellín	32 (78.0)	35 (81.4)	35 (81.4)	
Other municipalities	9 (22.0)	8 (18.6)	8 (18.6)	0.906*
Concomitant factors (%)				
Stress	34 (82.9)	29 (67.4)	30 (69.8)	0.227*
Sleeplessness	24 (58.5)	18 (41.9)	23 (53.5)	0.290*
Longer positions	27 (65.9)	34 (79.1)	29 (67.4)	0.342*
Muscles with trigger point (%)				
Trapezius muscle	37 (90.2)	42 (97.7)	39 (90.7)	0.326*
Levator scapulae	31 (75.6)	27 (62.8)	26 (60.5)	0.290*
Infraspinatus				
muscle	12 (29.3)	12 (27.9)	13 (31.0)	0.953*
Physical examination (%)				
Alteration sensitive	8 (19.5)	7 (16.3)	8 (18.6)	0.924*
Referred pain	22 (53.7)	36 (83.7)	23 (53.5)	0.004*
Local twitch response	29 (70.7)	26 (61.9)	22 (51.2)	0.183*
Visual Analogue				
Scale for Pain (Mean, SD)	63.5 (13.9)	68.7 (17.0)	64.2 (16.2)	0.340**
PHQ 9 (Mean, SD)	5.6 (3.2)	5.9 (4.2)	5.6 (3.5)	0.995 **
Quality of life SF-36 (Mean, SD)				
Bodily pain (BP)	38.8 (14.4)	35.2 (14.3)	38.5 (16.4)	0.642**
Role-emotional (RE)	66.4 (35.0)	75.8 (38.7)	67.2 (36.8)	0.273**
Role-physical (RP)	51.2 (40.0)	51.1 (37.7)	42.4 (37.6)	0.498**
Physical functioning (PF)	79.5 (17.8)	72.5 (20.5)	81.7 (11.9)	0.095**
Social function (SF)	69.5 (22.0)	68.0 (26.6)	71.8 (23.6)	0.799**
General health (GH)	67.4 (18.3)	57.3 (21.9)	70.7 (15.4)	0.01**
Mental health (MH	67.8 (14.2)	65.5 (18.9)	67.4 (17.5)	0.805**
Vitality (VT)	54.2 (13.9)	54.7 (19.2)	60.7 (19.3)	0.181**

*(Chi2)

**Kruskal-Wallis

Patient Health Questionnaire (PHQ-9), Items are scored from 0 (not at all) to 3 (nearly every day). Items are summed and the total score (from 0 to 27) represents the severity of depressive symptoms. Higher scores on the Visual Analogue Scale for Pain (VAS-P) indicate more pain, with a maximum of 100 (range, 0–100). Short Form Health Survey, the SF-36 with 8 sub-scales: *BP* bodily pain, *RE* role limitations as a result of emotional problems, *RP* role limitations as a result of physical problems, *PF* physical functioning, *SF* social functioning, *GH* general health perception, *MH* general mental health, *VT* vitality (the frequency of feeling full of energy vs feeling tired). Total sub-scale scores range from 0–100 with higher scores indicating better quality of life

All statistical analyses were conducted using an alpha of 0.05, so that a p value of less than 0.05 was considered to be statistically significant. Data analyses were performed using software SPSS® 18 (IBM, Armonk, New York).

## Results

Data was analyzed from 127 patients: 43 patients in the PT + LI group, 41 in the PT group, and 43 in the LI group. See the flowchart in Fig. 1.

A total of 92 % the of participants received public health care assistance and 10 % had hypertension. These proportions were similar among groups. Additional baseline characteristics of the three groups are shown in Table 1. Groups were similar in socio-demographic and clinical characteristics - 96 % had active trigger points and 4 % latent MTrPs at the time of examination, and 65 % of these experienced a characteristic pattern of referred pain. A significantly larger proportion of the PT group (83.7 %) had referred pain compared to the other two groups (53 % each one; *p* < 0.01). Functional limitations presented by the patients were as follows: 15 % could not lower horizontal objects, 14 % had difficulty combing their hair, 9 % had difficulty dressing, and 6.5 % had difficulty bathing. These proportions of patients experiencing functional limitations were similar across the three groups.

In terms of the limitation in range of motion, the head tilt to the left was the most limited (31 %) followed by the right slant (27 %), rotations to the left (19 %) and right (14 %), flexion (7.3 %) and finally extension (5.6 %). There were no significant differences between the groups in range of motion.

At baseline, patients in each group had similar VAS pain scores, similar PHQ-9 scores as a measure of depressive symptoms, and similar QoL on the all the dimensions of the SF-36 except in the general health domain in which those in the PT group reported significantly worse general health QoL compared to the PT + LI and LI groups [57.3 (21.9) vs. 67.4 (18.3) and 70.7 (15.4), respectively, *p* = 0.01].

### Primary outcome

As shown in Table 2, there were no statistically significant differences in pain ratings at 1 month as measured by the VAS between the PT + LI group [40.8 (25.3)] and the PT group [37.8 (21.9), *p* = 0.56] or the LI group [44.2 (24.9), *p* = 0.545].

**Table 2:** Primary and secondary outcomes in the Physical Therapy and Local injection, Physical Therapy + Local injection, Physical Therapy and Local injection at 1 month in patients with myofascial pain syndrome

Outcomes	Physical therapy + local injection (*n* = 41)	Physical therapy (*n* = 43)	*P* value*	Local injection (*n* = 43)	*P* value*
Visual Analogue Scale for Pain (Mean, SD)	40.8 (25.3)	37.8 (21.9)	0.560	44.2 (24.9)	0.545
PHQ 9 (Mean, SD)	4.33 (3.65)	4.43 (4.073)	0.904	4.44 (3.03)	0.879
Quality of life SF-36 (Mean, SD)					
Bodily pain (BP)	55.3 (18.4)	57.12 (20.70)	0.89	51.78 (15.70)	0.29
Role-emotional (RE)	78.2 (35.1)	85.95 (27.58)	0.31	78.76 (33.24)	0.94
Role-physical (RP)	73.1 (40.5)	73.84 (34.48)	0.56	74.39 (33.30)	0.58
Physical functioning (PF)	86.8 (13.2)	82.79 (15.52)	0.21	86.83 (11.55)	0.72
Social function (SF)	79.2 (20.7)	81.51 (22.83)	0.38	78.15 (20.44)	0.78
General health (GH)	72.1 (16.7)	65.70 (20.16)	0.12	73.54 (13.43)	0.99
Mental health (MH	73.2 (16.6)	70.05 (19.82)	0.46	69.95 (17.57)	0.34
Vitality (VT)	61.3 (16.0)	59.88 (19.83)	0.66	62.56 (17.32)	0.95

*T-test to compare differences between groups at month

Patient Health Questionnaire (PHQ-9), Items are scored from 0 (not at all) to 3 (nearly every day). Items are summed and the total score (from 0 to 27) represents the severity of depressive symptoms. Higher scores on the Visual Analogue Scale for Pain (VAS-P) indicate more pain, with a maximum of 100 (range, 0–100). Short Form Health Survey, the SF-36, with 8 sub-scales: *BP* bodily pain, *RE* role limitations as a result of- emotional problems, *RP* role limitations as a result of physical problems, *PF* physical functioning, *SF* social functioning, *GH* general health perception, *MH* general mental health, *VT* vitality (the frequency of feeling full of energy vs. feeling tired). *SF* social functioning, *MH* general mental health. Total sub-scale scores range from 0–100 with higher scores indicating better quality of life

### Secondary outcomes

At 1 month, there were also no significant differences between groups in their PHQ-9 depression scores or on any of the quality of life dimensions measured by the SF-36 sub-scales (see Table 2). Individual SF-36 item differences between groups were analyzed. The only item in which there was a significant difference was the change in health item in which the PT + LI group had significantly higher QoL [70.0 (18.1)] compared to the PT group [60.93 (18.5), *p* = 0.03]. The LI group mean on this item was 67.8 (16.1) and not significantly different from the PT + LI group mean (*p* = 0.49).

The 3-month outcomes are shown in Table 3. There were no statistically significant differences between groups in VAS pain scores, PHQ-9 depression scores, or SF-36 quality of life sub-scales.

**Table 3:** Secondary outcomes in the physical therapy and local injection, physical therapy + local injection, physical therapy and local injection at 3 months in patients with myofascial pain syndrome

Outcomes	Physical therapy + local injection (*n* = 41)	Physical therapy (*n* = 43)	*P* value	Local injection (*n* = 43)	*P* value*
Visual Analogue Scale for Pain (Mean, SD)	21.6 (21.8)	28.2 (23.7)	0.192	28.8 (22.3)	0.141
PHQ 9 (Mean, SD)	3.98 (3.97)	4.12 (4.48)	0.877	3.16 (2.81)	0.284
Quality of life SF-36 (Mean, SD)					
Bodily pain (BP)	66.58 (20.09)	65.66 (20.9)	0.69	61.19 (17.67)	0.16
Role-emotional (RE)	83.25 (32.11)	84.13 (32.95)	0.70	90.60 (21.15)	0.38
Role-physical (RP)	81.88 (30.48)	82.5 (31.11)	0.75	87.79 (22.07)	0.44
Physical functioning (PF)	87.75 (16.94)	89.13 (12.29)	0.56	90 (9.06)	0.41
Social function (SF)	82.6 (20.99)	83.05 (20.71)	0.88	83.28 (17.44)	0.87
General health (GH)	71.67 (17.26)	68.25 (22.37)	0.72	77.44 (12.50)	0.15
Mental health (MH	75.3 (16.79)	71.32 (17.86)	0.30	78.95 (14.03)	0.43
Vitality (VT)	63.38 (16.11)	61.95 (18.53)	0.77	69.64 (16.17)	0.20

*T-test to compare differences between groups at month

Patient Health Questionnaire (PHQ-9), Items are scored from 0 (not at all) to 3 (nearly every day). Items are summed and the total score (from 0 to 27) represents the severity of depressive symptoms. Higher scores on the Visual Analogue Scale for Pain (VAS-P) indicate more pain, with a maximum of 100 (range, 0–100). Short Form Health Survey, the SF-36 with eight sub-scales: *BP* bodily pain, *RE* role limitations as a result of- emotional problems, *RP* role limitations as a result of physical problems, *PF* physical functioning, *SF* social functioning, *GH* general health perception, *MH* general mental health, *VT* vitality (the frequency of feeling full of energy vs. feeling tired), *SF* social functioning, *MH* general mental health. Total sub-scale scores range from 0–100 with higher scores indicating better quality of life

Regarding function, at 1 month and 3 months, the PT and PT + LI groups showed higher right upper limb hand-back maneuver scores compared to the LI alone group (*p* = 0.013 and *p* = 0.016 respectively). The left upper limb hand-back maneuver and right and left hand-mouth maneuvers were not significantly different between groups.

### Analgesic use and complications

There were no significant differences in analgesic use between groups (*p* = 0.10); 36.6 % of the PT + LI group used took analgesics, while 53.0 % of the PT group and 51.2 % of the LI group used these supplementary medications.

There were complications documented for six patients. In the PT + LI group, four participants had localized hematomas. In the LI group, two participants had localized hematomas and one participant had minimal bleeding. In total, for the number of injections performed (*n* = 84) the complication rate was 2.66 %. There were no complications in the PT group.

## Discussion

In this study, the efficacy and safety of two interventions, PT and LI, were compared, alone vs. in combination, in male and female patients over 18 years of age with shoulder or neck pain of more than 6 weeks duration and MTrPs of the trapezius, infraspinatus, and/or levator scapulae (cervical portion). There were no statistically significant differences between the combination group and the individual treatment alone groups in VAS pain ratings, PHQ-9 depression scores, or SF-36 QoL domains at neither 1 nor 3 months.

There are no studies in the literature that evaluate head-to-head whether lidocaine injection into MTrPs combined with a PT program might be more effective than each separate treatment alone in improving pain, function, and quality of life in patients with MPS of the shoulder girdle and cervical region. However, in a recent Cochrane review of treatments for chronic neck pain, moderate quality evidence supports cervico-scapulothoracic and upper extremity strength and endurance exercises and combined cervical, shoulder and scapulothoracic strengthening and stretching exercises to improve pain and function [36]. The present study’s PT treatment incorporated similar PT exercises, without homework exercises, and included patients with MTrPs in the infraspinatus muscle as well. In this study, PT alone and in combination with LI showed better right, but not left limb hand-back maneuver functioning compared to LI injection alone. Several studies have documented the effectiveness of PT exercises on myofascial pain. For instance, an RCT of patients with MTrPs in the upper trapezius found that employing different manual techniques (such as ischemic compression, stretching after ischemic compression, and passive stretching) resulted in improved range of motion and pain sensitivity; outcomes in this study were measured only at 24 h and 1 week, and other functional outcomes were not evaluated [37]. In this study, PT treatment involved the use of hot packs and ultrasound, manual compression, deep stroking or strumming, and muscle-stretching and strengthening exercises. These techniques were focused on the cervical and shoulder girdle and specifically the trapezius, infraspinatus, and the levator scapulae muscles. Furthermore, the current study assessed longer term outcomes at 1 and 3 months post-treatment, and the groups receiving PT had significantly better right upper limb hand-back maneuver function. However, left upper limb function was not significantly different between groups, so additional research on the value of PT focused on these muscles in this patient group may be warranted. Another study in patients with shoulder pain of more than 6 months with a MTrP average pain rating of 7.4 (3.6) showed that a comprehensive PT treatment once a week with manual TP compression, stretching, cold application, and instructions for home exercises was more effective than doing nothing to improve the function; this improvement was demonstrated on the DASH (Disabilities of the Arm, Shoulder, and Hand) and on pain measured via the VAS at the time of the evaluation and in the final 7 days of treatment [29]. The present study PT treatment incorporated similar PT exercises and also included patients with MTrPs in the shoulder (infraspinatus muscle) but we did not use the DASH Scale to measure function.

Massage, another technique incorporated into the PT treatment group of the present study, has been shown to be effective in reducing pain compared with inactive therapies, such as usual care; however, massage alone has not been found to be more effective than active therapies, such as exercise [23]. In one study, massage was effective for the relief of subacute and chronic neck pain, but only in the short term [38]. In the present study, massage was used, but only in combination with other PT techniques, i.e., muscles involved were stretched and strengthened, thus further study that isolates the effects of massage alone on function or other outcomes may prove valuable.

Regarding lidocaine injections, in this study, using lidocaine injections alone was found to be similarly effective to lidocaine plus physical therapy. Similarly, in an RCT conducted with a smaller sample size of 80 patients, both dry needling and an injection of lidocaine improved pain ratings, but there were no differences between the groups [39]. In the present study, lidocaine was injected only one time and it is possible that multiple injections over time might lead to more improvement. One study showed that, compared to placebo, a series of 5 lidocaine injections on alternating days to the pericranial muscles significantly reduced both the frequency and severity of pain at 2, 4 and 6 months post treatment in MPS patients. [40] Additional RCTs examining the intensity and frequency of the proposed interventions, either alone or in combination, may help guide clinicians in their treatment strategies.

In a systematic review of needling therapies in the management of MTrP pain, the effect of MTrP injections was independent of the injected substance, which in most studies was an anesthetic such as lidocaine, bupivacaine, or procaine. No trials were of sufficient quality or design to test the efficacy of any needling technique beyond placebo in the treatment of myofascial pain. In another systematic review with meta-analysis, there was no significant difference between dry needling and lidocaine in the management of MTrPs in the neck and shoulder region. The quality of evidence for the studies was limited [41].

In another study, TrP pain pressure values were significantly higher in lidocaine group than dry needle and pain scores were significantly lower than in both the botulinum toxin type A (BTX-A) and dry needle groups. The Canadian Agency for Drugs and Technologies for Health (CADTH) conducted a Rapid Response and synthesized evidence from identified one systematic review and five RCTs. They concluded that evidence cannot support the use of botulinum toxin type A (BoNTA) injection in MTrPs for myofascial pain, given that four out of five included trials reported that BoNTA had no statistically significant effect on pain. Therefore, BoNTA was not considered to be effective for the treatment of MPS [42]. In summary, there are few outcome studies of good quality and, although some studies showed reduction in pain scores and pressure pain thresholds, the literature has neither convincingly supported nor refuted the effectiveness of some invasive and non-invasive modalities beyond placebo [13].

### Study limitations

There are a number of limitations of the present study. Because there were three different physical therapists who conducted the PT intervention, it is possible that inter-therapist effects may have influenced the findings. However the PT sessions were standardized via a protocol to minimize this potential confounder. Similarly, there were various medical specialists who performed the injections, but all had more than 5 years of experience in the field with MPS patients. Additionally, no validated functional measure centered on the patient, such as the DASH, was used. Future studies should include a validated measure of function and a measure of the impact of the condition on work performance, as well as an evaluation related to patient occupation and physical activity. In the current study, patients were only followed for 3 months, and additional research with longer follow-ups and a more comprehensive evaluation of outcomes is needed to assess how much of the improvement is maintained, the likelihood of relapses, and the need for further consultation and treatment. Finally, it is known that studies of the efficacy of MTrPs interventions have shown marked statistical heterogeneity that make it is difficult to evaluate outcomes [43]. Therefore, it is necessary to conduct further studies with a larger number of patients so that analyses can examine different subgroups of patients and determine if the interventions might help to treat underlying pathologies, including spinal conditions, postural abnormalities, and underlying behavioral issues. Furthermore, it is important to include patients on a waiting list or in a sham treatment placebo group in order to assess the superiority of one intervention over another in this patient population [44].

## Conclusions

Myofascial pain is often chronic and treatment with any of the interventions studied produced similar outcomes, except for a slight advantage of those receiving interventions with PT showing better right limb hand-back maneuvering function compared to the LI only group. These findings suggest that PT treatment involving the use of hot packs and ultrasound, manual compression, deep stroking or strumming, and muscle-stretching and strengthening exercises could play a role in improving upper limb function, although more research is needed. Lidocaine injections, which produced similar outcomes in the MPS patients studied, are an inexpensive treatment even in repeated over time. Other studies have found that botulinum toxin is no more effective than injections with local anesthetic and is more expensive [42, 45–47]. Future research should examine more specific PT treatments at varying time intervals, conduct longitudinal assessment of more comprehensive longer-term outcomes, assess and control for contextual factors like the patient’s occupation, and incorporate additional outcomes such as relapses and disability.

### Ethics approval and consent to participate

Approval was obtained from the Ethics Committee of the University of Antioquia. All participants provided informed consent.

### Consent for publication

Not applicable.

### Availability of data and materials

The dataset supporting the conclusions of this article is available in the gruporehabilitacionsalud@udea.edu.co in the University of Antioquia.

## Abbreviations

GRADE: Grading of Recommendations Assessment, Development, and Evaluation
LI: lidocaine injections
MPS: myofascial pain syndrome
MTrPs: myofascial trigger points
PHQ – 9: patient health questionnaire – 9
PT: physical therapy
RCTs: randomized controlled trials
SF36: short form – 36
VAS: visual analog scale
